# Point of Care Ultrasound (POCUS) Used to Rapidly Diagnose Both Renal Colic and a Symptomatic Abdominal Aortic Aneurysm in an Elderly Man with Left Flank Pain

**DOI:** 10.24908/pocusj.v10i01.18461

**Published:** 2025-04-15

**Authors:** Rie Seu, Ariella Gartenberg, Rachel Mirsky, Aamir Bandagi, Nicole J. Leonard-Shiu, Reema Panjwani, Nora McNulty, Trevor Dixon, Michelle A. Montenegro, Michael Halperin

**Affiliations:** 1 Department of Emergency Medicine, Albert Einstein College of Medicine - NYC Health + Hospitals Corporation Jacobi Medical Center, Bronx, NY, USA; 2 Department of Emergency Medicine, Albert Einstein College of Medicine - Montefiore Medical Center, Bronx, NY, USA; 3 Department of Emergency Medicine, St. Barnabas Hospital, Bronx, NY, USA; 4 Department of Emergency Medicine, Vanderbilt University, Nashville, TN, USA

**Keywords:** abnormal aortic aneurysm, abdominal pain, nephrolithiasis, point of care ultrasound, POCUS

## Abstract

Abdominal aortic aneurysms (AAAs) possess significant patient morbidity and mortality, but diagnosis can be missed or delayed given variable presenting symptoms. Renal colic is a potential “red herring” in cases of symptomatic AAA. This case involves an atypical presentation of flank pain likely due to both nephrolithiasis and an AAA. Prompt recognition of AAAs by the emergency department (ED) is critical to prevent misdiagnosis and initiate rapid treatment when indicated.

## Case Report:

**Figure 1.**
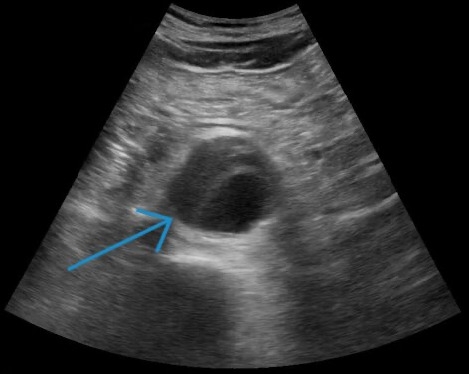
Point of care ultrasound (POCUS) demonstrating a 5.0 x 5.3 cm abdominal aortic aneurysm.

**Figure 2.**
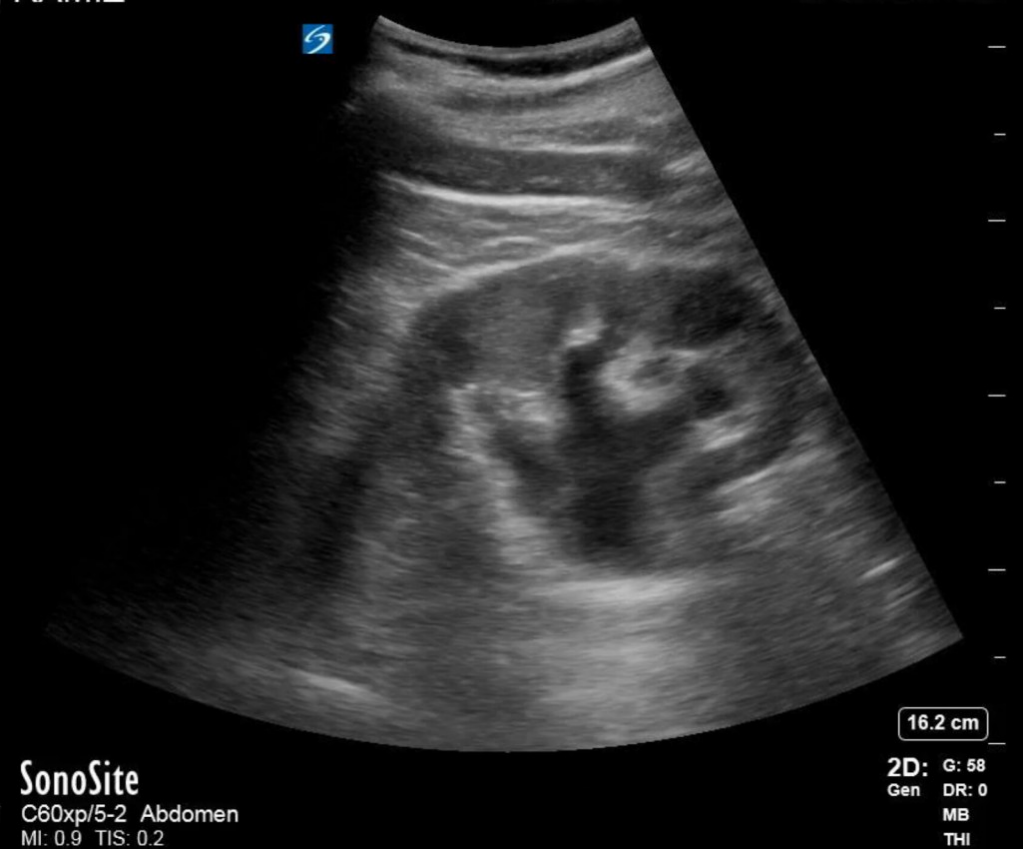
Renal point of care ultrasound (POCUS) demonstrating moderate left-sided hydronephrosis without surrounding free fluid.

**Figure 3.**
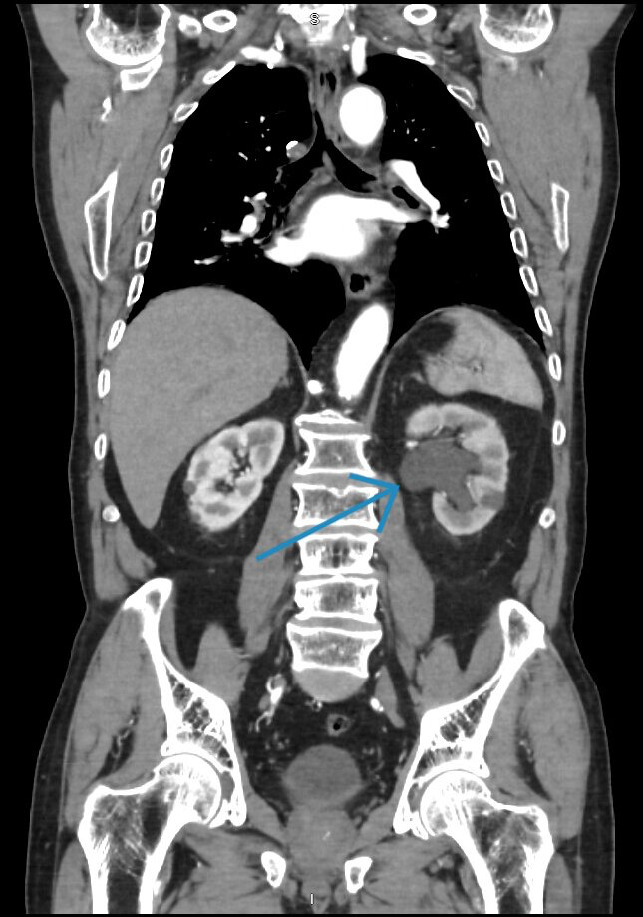

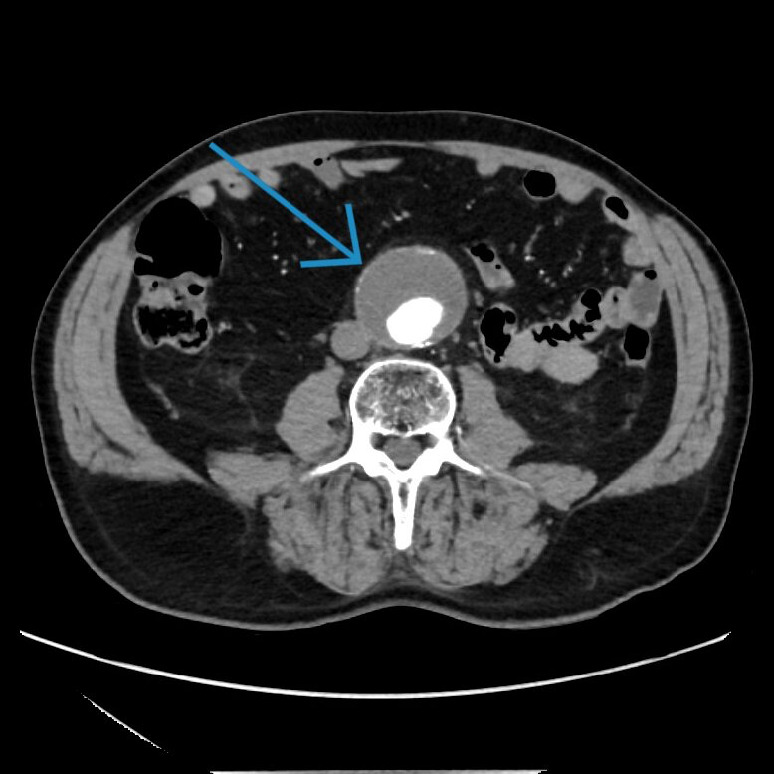
Computed tomography (CT) angiogram of the chest, abdomen, and pelvis demonstrating a 5 x 5.3 cm infrarenal aortic aneurysm extending to the aortic bifurcation (8.9 cm long) and a 1.3 cm calculus at the left ureteropelvic junction, resulting in moderate hydronephrosis.

A 70-year-old man with a history of tobacco use, hypertension, nephrolithiasis, chronic systolic heart failure, and recent diagnosis of a 5 cm infrarenal AAA one-month prior presented to the emergency department (ED) with acute onset left flank and lower back pain for two weeks. The pain was progressively worsening, constant, and 10/10 on maximal intensity. No associated symptoms were reported. Initial vital signs were a blood pressure of 143/76 mm Hg, heart rate 59 bpm, temperature 97.3 F, and oxygen saturation 93% on room air. The physical examination was notable for left-sided costovertebral angle tenderness without overlying skin changes. No abdominal tenderness, rebound, or guarding was present. Laboratory investigations revealed a white blood cell count of 8.31 and hemoglobin/hematocrit 15.2/45.6. Urine analysis showed no bacteria, positive red blood cells, negative leukocyte esterase, and negative nitrites. Urine culture eventually resulted with no growth. Aortic and renal point of care ultrasound (POCUS) were promptly performed by the emergency physician. Aortic POCUS showed an abdominal aortic aneurysm (AAA) measuring 5 x 5.3 cm (Figure 1) and extending 8.9 cm long to the aortic bifurcation. No free fluid was visualized in the right upper quadrant. Renal POCUS demonstrated left-sided moderate hydronephrosis concerning for unilateral ureteral obstruction (i.e., from presumed nephrolithiasis) (Figure 2). The patient continued to endorse severe back pain that was unrelieved with repeat narcotics. The vascular surgery and urology teams were immediately notified and computed tomography (CT) angiogram of the chest, abdomen pelvis was performed. The CT scan now revealed a 5 x 5.3 cm infrarenal aortic aneurysm extending to the aortic bifurcation (8.9 cm long) and a 1.3 cm calculus at the left ureteropelvic junction resulting in moderate hydronephrosis (Figure 3). With progression of the AAA and uncontrolled lower back pain, urgent repair was recommended by the vascular team. The patient underwent successful endovascular aneurysm repair, with resolution of the lower back pain, but not of his left sided flank pain. The patient later underwent retrograde ureteroscopy with laser lithotripsy and nephrostomy tube placement. Complete resolution of the flank pain was achieved.

## Discussion

### Abdominal Aortic Aneurysm (AAA) and Obstructing Nephrolithiasis

An aneurysm is a segmental, full-thickness vascular dilation [1]. While normal diameter can vary with age, gender, and weight, the American College of Radiology (ACR), the American Institute of Ultrasound in Medicine (AIUM), the Society for Pediatric Radiology (SPR), and the Society of Radiologists in Ultrasound (SRU) guidelines define an infrarenal AAA as “greater than or equal to 3 cm in diameter or greater than or equal to 1.5 times the diameter of the more proximal infrarenal aorta” [1]. The abdominal aorta is the most common site of arterial aneurysms. Risk factors include smoking, male gender, advanced age, atherosclerosis, connective tissue disorders, prior arterial aneurysms, and prior aortic dissection or aortic surgery [2]. The risk of rupture is increased in females and current smokers, as well as patients with uncontrolled hypertension, rapid aortic expansion (greater than 0.5 cm per year), and large initial AAA diameter (greater than 5.5 cm) [2,3].

AAAs can present in a variety of ways. The majority of patients are asymptomatic, with AAAs discovered incidentally on imaging, during screening, or on physical exam by the presence of a pulsatile mass (as seen in 30% of patients) [4]. When patients are symptomatic, they typically present with flank, abdominal, or back pain [5]. Patients may also present with signs or symptoms of limb ischemia due to thrombus embolism or atherosclerotic debris. The triad of symptoms—acute severe pain, pulsatile abdominal mass, and hypotension—is only present in 26% of patients with ruptured AAAs [6].

Progressive or symptomatic AAAs can be underdiagnosed, with renal colic/nephrolithiasis as a potential “red herring” [7,8]. Given that AAAs can rapidly enlarge or progress to rupture, a high index of suspicion is critical to avoid rapid deterioration and aide in timely recognition. POCUS can be used to rapidly evaluate an AAA, as can ultrasound or CT of the abdomen.

In one systematic review including 655 patients, POCUS can identify patients with symptomatic AAAs presenting to the ED with a sensitivity of 99% and specificity of 98% [9]. Additionally, POCUS has been shown to detect hydronephrosis in patients presenting to the ED with flank pain with an overall sensitivity and specificity of 86.8 (95% confidence interval [CI] = 78.8 to 92.3) and 82.4 (95% CI = 74.1 to 88.1), respectively [10]. Sensitivity and specificity were increased in patinets with both flank pain and hematuria [10].

POCUS for patients with symptomatic AAAs differ from POCUS utilization for screening purposes. While high rates of detection are present in symptomatic patients, POCUS for AAA screening purposes has demonstrated a high false positive rate approaching 21.4% by family physicians in public primary healthcare settings [11]. Despite high false positive rates, early identification and surgical intervention can reduce patient morbidity and mortality. A reduction in mortality from ruptured AAA since 1997 in England and Wales was attributed to changes in cigarette smoking and increases in elective AAA repair in patients over 75-years-old [12]. Mortality from ruptured AAA in patients over 65-years-old decreased from 65.9 to 44.6 per 100,000 individuals [12].

In the case discussed, clinical examination and POCUS were paramount to avoid assuming the symptomatic nephrolithiasis was the only cause of the patient's symptoms. Re-evaluation of the patient's symptoms, notably his uncontrolled lower back pain, in combination with his known AAA, were critical in this case. Distinguishing between pain associated with nephrolithiasis and symptomatic AAA can be difficult, and in this case both pathologies likely contributed to the patient's discomfort. This case serves as a reminder that Hickam's Dictum—the notion that “patients can have as many diseases as they damn well please”—may occasionally prevail over Occam's Razor, the rule of diagnostic parsimony [13].
